# Anlotinib suppresses lymphangiogenesis and lymphatic metastasis in lung adenocarcinoma through a process potentially involving VEGFR-3 signaling

**DOI:** 10.20892/j.issn.2095-3941.2020.0024

**Published:** 2020-08-15

**Authors:** Tingting Qin, Zhujun Liu, Jing Wang, Junling Xia, Shaochuan Liu, Yanan Jia, Hailin Liu, Kai Li

**Affiliations:** ^1^Department of Thoracic Oncology, Tianjin Medical University Cancer Institute and Hospital, National Clinical Research Center for Cancer; Key Laboratory of Cancer Prevention and Therapy, Tianjin; Tianjin’s Clinical Research Center for Cancer, Tianjin 300060, China; ^2^Department of Biomedical Engineering, Tianjin Medical University, Tianjin 300070, China

**Keywords:** Anlotinib, VEGFR-3 dephosphorylation, lymphangiogenesis, lymph node metastasis, lung adenocarcinoma

## Abstract

**Objective:** Lymphatic metastasis is one of the leading causes of malignancy dispersion in various types of cancer. However, few anti-lymphangiogenic drugs have been approved for clinical use to date. Therefore, new therapies to block lymphangiogenesis are urgently required.

**Methods:** Immunohistochemistry, immunofluorescence, Western blot, migration assays, and lymphangiogenesis and lymphatic metastasis assays were used.

**Results:** Anlotinib, a receptor tyrosine kinase inhibitor, suppressed the rate of new metastatic lesions (31.82% in the placebo arm and 18.18% in the anlotinib arm) in patients with advanced lung adenocarcinoma who were enrolled in our ALTER-0303 study. D2-40^+^-lymphatic vessel density was strongly correlated with disease stage, metastasis, and poor prognosis in 144 Chinese patients with lung adenocarcinoma. In mice bearing A549^EGFP^ tumors, tumor lymphatic vessel density, tumor cell migration to lymph nodes, and the number of distant metastatic lesions were lower in the anlotinib group than in the controls. Anlotinib inhibited the growth and migration of human lymphatic endothelial cells (hLECs) and lymphangiogenesis *in vitro* and *in vivo*. Treatment of hLECs with anlotinib downregulated phosphorylated vascular endothelial growth factor receptor 3 (VEGFR-3).

**Conclusions:** Anlotinib inhibits lymphangiogenesis and lymphatic metastasis, probably through inactivating VEGFR-3 phosphorylation. The results indicate that anlotinib may be beneficial for treatment in avoiding lymphangiogenesis and distant lymphatic metastasis in lung adenocarcinoma. (Trial registration: ALTER0303; NCT02388919; March 17, 2015.)

## Introduction

Lung cancer is the leading cause of cancer-related death (18.4% of total cancer deaths) worldwide^1^. Metastasis is responsible for approximately 90% of cancer deaths^2^. The lymphatic system plays an important role in tumor metastasis^3^. Non-small cell lung cancer (NSCLC) tumor cells usually appear in regional lymph nodes before they are detected in distant organs. The presence of lung cancer cells in lymph nodes is a major indicator in the staging of NSCLC and is associated with poor prognosis^4,5^. Tumor lymphangiogenesis is strongly correlated with increased incidence of lymph node metastasis and decreased overall survival in several types of human cancers^6–8^ and experimental cancer models^9–11^. Improving understanding of tumor lymphangiogenesis would contribute to the development of effective therapeutic strategies.

Unfortunately, some clinical studies have reported that anti-angiogenic therapy facilitates lymphatic metastasis *via* prompting compensatory lymphangiogenesis in tumors^12,13^. Among the signaling pathways important in regulating lymphangiogenesis in pathological situations^14^, vascular endothelial growth factor receptor 3 (VEGFR-3) signaling plays a central role^15^. Therefore, targeting the VEGFR-3 signaling axis is a potential strategy to prevent or decrease lymphatic metastasis. Although many drugs targeting VEGFR-3 are available, including axitinib, cabozantinib, lenvatinib, sorafenib, sunitinib, and pazopanib^16^, there are no effective drugs targeting lymphangiogenesis for clinical use.

Anlotinib (also known as AL3818), which has been approved in China as a third-line treatment option for patients with advanced NSCLC^17–19^, is a novel oral multi-target receptor tyrosine kinase inhibitor that inhibits VEGFR-1, VEGFR-2, VEGFR-3, c-Kit, c-Met, c-Src, PDGFRβ, HER2, and EGFR. It has been found to have higher inhibitory activity against VEGFR-2 (0.2 ± 0.1 nmol/L) and VEGFR-3 (0.7 ± 0.1 nmol/L) than other targets in a preclinical study^20^. VEGFR-2 is expressed primarily in blood vascular endothelial cells, whereas VEGFR-3 is expressed in lymphatic endothelial cells^21^. Anlotinib specifically inhibits angiogenesis in tumors by suppressing the activation of VEGFR-2^20,22^. However, the effects of anlotinib on VEGFR-3, lymphangiogenesis and new metastatic lesions in lung adenocarcinoma remain unclear.

In this study, first we report that the density of lymphatic vessels in lung adenocarcinoma tumor tissues strongly correlates with disease progression and poor prognosis in 144 patients with lung adenocarcinoma. In our previous ALTER-0303 study, we found that anlotinib suppressed the formation of new metastatic lesions in 66 patients with advanced lung adenocarcinoma. Second, we show that anlotinib has a critical effect on lymphangiogenesis, and lymphatic metastasis in mouse models of lung adenocarcinoma. Third, we report that anlotinib inhibits the growth and migration of human lymphatic endothelial cells (hLECs) and lymphangiogenesis *in vitro* and *in vivo*. Finally, we demonstrate that anlotinib disrupts the phosphorylation of VEGFR-3, thus leading to decreased lymphangiogenesis, and describe the possible underlying molecular mechanism. These findings provide new insights into anlotinib’s role in lymphangiogenesis and abrogating lymph node metastasis in lung cancer. Our study sheds light on the promising therapeutic potential of anlotinib in the treatment of tumor lymphatic metastasis.

## Materials and methods

### ALTER0303 clinical trial protocol

The ALTER 0303 trial was a multicenter, double-blind, phase 3 randomized clinical trial designed to evaluate the efficacy and safety of anlotinib in patients with advanced NSCLC. Patients from 31 grade-A tertiary hospitals in China were enrolled between March 1, 2015, and August 31, 2016. Patients 18–75 years of age who had histologically or cytologically confirmed NSCLC were eligible (*n* = 606), and those who had centrally located squamous cell carcinoma with cavitary features or brain metastases that were uncontrolled or controlled for less than 2 months were excluded. Patients (*n* = 440) were randomly assigned in a 2-to-1 ratio to receive either 12 mg/day of anlotinib or a matched placebo. All patients were treated with study drugs at least once in accordance with the intention-to-treat principle^18^.

### Clinical specimens

The Ethics Committee of Tianjin Medical University Cancer Institute and Hospital (Tianjin, China) approved the use of human tissues for this study (EK2018039). Each patient signed an informed consent form for participation. All 66 patients enrolled in the ALTER-0303 study in our center were documented as having advanced lung adenocarcinoma at Tianjin Medical University Cancer Institute and Hospital between 2015 and 2016. Primary lung adenocarcinoma tissues were collected from 144 patients who underwent surgery at the Tianjin Medical University Cancer Institute and Hospital between 2011 and 2016. All specimens were confirmed independently by 2 experienced pathologists.

### Animals and subcutaneous lung cancer models

Female 6-week old BALB/c-nu or C57BL/6J mice were purchased from the Model Animal Center of Nanjing University. All experimental procedures were performed in accordance with protocols approved by the Institutional Animal Care and Research Advisory Committee of Tianjin Medical University. To induce a subcutaneous Lewis lung cancer (LLC) model, LLC cells were inoculated (5 × 10^6^ cells/mouse) in the subcutaneous space near the axillary lymph nodes in C57BL/6J mice. The mice were then randomized to 2 groups treated with anlotinib or saline (3 mg/kg orally daily for 21 days, as previously described)^23^. The control group was treated with aseptic saline, and the anlotinib group was treated with anlotinib dissolved in aseptic saline. Tumor sizes were measured with dial calipers every other day in a blinded manner. Tumor volumes were determined with the equation: volume = 0.52 × length × width × width. The mice were euthanized at the end of the experiment. Tumors and inguinal lymph nodes were retrieved for pathologic analyses. A549^EGFP^ cells were used to determine the effect of anlotinib on tumor-associated lymphatic metastasis of human lung cancer cells. A549^EGFP^ cells were generated in our laboratory from A549 cells. A549^EGFP^ cells were inoculated (5 × 10^6^ cells per mouse) in the subcutaneous space near the axillary lymph nodes in nude mice. The research methods were as described above. Metastatic lesions were imaged with an IVIS Spectrum Imaging System (Caliper Life Science, Hopkinton, MA, USA), which enabled visualization of A549^EGFP^ tumors in intact mice. Despite the emission of auto-fluorescence signals in naive mice, high intensity fluorescence signals from A549^EGFP^ tumors were distinguishable and were normalized to the background signal.

### Cells

LLC and A549 cells were purchased from ATCC (Manassas, VA, USA), and A549^EGFP^ cells were generated in our laboratory from A549 cells as previously described^9^. These cells were maintained in 10% FBS RPMI 1640 (Gibco, Waltham, MA, USA). hLECs were purchased from ScienceCell and cultured in 10% FBS endothelial cell medium (ECM, ScienceCell Research Laboratories, Carlsbad, CA, USA).

### Reagents

Anlotinib was a gift from Jiangsu Chia-Tai Tianqing Pharmaceutical Co., Ltd. Antibodies against D2-40, podoplanin, LYVE-1, VEGFR-3, and Ki67 were purchased from Abcam (Cambridge, London, UK). Antibodies against p-Akt, Akt, p-Erk, Erk, VEGFR-2, p-VEGFR-2, and β-actin, as well as HRP-conjugated secondary antibodies were from CST (Danvers, MA), and antibody against p-VEGFR-3 (Tyr1230/1231) was from Cell Applications Inc. Alexa Fluor 555-conjugated secondary antibody was from Invitrogen Corporation (Carlsbad, CA). Freund’s incomplete adjuvant (IFA) was from Sigma-Aldrich (St Louis, MO). Human VEGF-C, a cytokine, was purchased from R&D Systems (Minneapolis, MN).

### Immunohistochemistry and immunofluorescence assays

Five-micrometer sections of formalin-fixed, paraffin-embedded tumors of clinical specimens, experimental tumors models, and lymph nodes were deparaffinized in xylene and rehydrated in a graded alcohol series, then rinsed with PBS. For antigen retrieval, sections were microwaved in citric acid solution (pH 6.0) for 15 min. Then, the slides were incubated in 3% H_2_O_2_ for 15 min. For immunohistochemical staining, the slides were incubated with antibodies against D2-40, podoplanin, or Ki67, then with appropriate secondary antibodies at RT for 30 min. Counterstaining was performed with hematoxylin for 2 min. Three clinical pathologists assessed the intensity of immunostaining in each section independently in a blinded manner. At least 10 fields/specimen were surveyed. For immunofluorescence staining, LYVE-1 and DAPI were used. The slides were incubated at RT for 1 h in the dark. Immunostained sections were imaged with a positive fluorescence microscope (Carl Zeiss, Oberkochen, Germany). At least 10 fields per section were analyzed.

### Western blot analyses

Total protein was extracted from homogenized hLECs in RIPA buffer and subjected to SDS-PAGE, transferred to PVDF membranes (Roche Molecular Biochemicals, Quebec, Canada), blocked with 5% BSA for 1 h at RT, and then immunoblotted overnight at 4 °C with the appropriate primary antibodies against target proteins. The blots were further incubated with HRP-conjugated secondary antibodies and developed with the ECL System (Millipore, Billerica, MA).

### *In vivo* neolymphangiogenesis assay

Female 6-week old BALB/C mice were randomized to 2 groups and injected with 200 μL IFA (1:1 mixed with PBS) intraperitoneally on day 0 and 14, respectively. Mice were treated with anlotinib or saline (3 mg/kg orally daily for consecutive 15?days) from day 10. Lymphangiomas developed by day 25^24^. The liver together with lymphangiomas was dissected, fixed and immunostained for lymphatic vessels, as described previously^25^.

### *In vitro* lymphangiogenesis assay

hLECs were plated in 48-well plates coated with 100 μL Matrigel (R&D Systems) per well for 6 h. Then cells were stained with 3 mM calcein-AM (Invitrogen). Formation of capillary tubule structures was observed and digitally photographed under an inverted light microscope at 5× magnification (Axiovert 200M; Zeiss, Oberkochen, Germany). Tube lengths and areas were quantified in Image-Pro Plus 6.0 software (Media Cybernetics, Rockville, MD, USA).

### hLEC migration assays

hLECs were seeded in the upper compartment of Transwell inserts coated with 0.2% gelatin, at 1.5 × 10^5^/insert in EBM-2 medium containing 0.5% FBS, and treated with anlotinib or saline. The lower compartment was filled with EBM-2 containing 1% FBS and VEGF-C (400 ng/mL). After 24 h incubation, the inserts were rinsed, fixed, and stained with crystal violet (Beyotime, Haimen, Jiangsu, China). The number of cells that migrated per field were counted in 6 randomly chosen fields per well acquired at 200× magnification (ECLIPSE Ti, Nikon, Tokyo, Japan).

### Statistical analysis

Data were subjected to variance analysis (ANOVA) followed by 2-tailed, unpaired Student’s *t*-test. The Spearman correlation rank for nonparametric variables was used to assess the relationships between categorical variables. Survival curves were calculated with the Kaplan-Meier method, and the differences were estimated with the log-rank test. Differences with *P*-values less than 0.05 were considered statistically significant.

## Results

### Anlotinib inhibits new metastatic lesions in 44 patients with advanced lung adenocarcinoma

**Figure 1:**
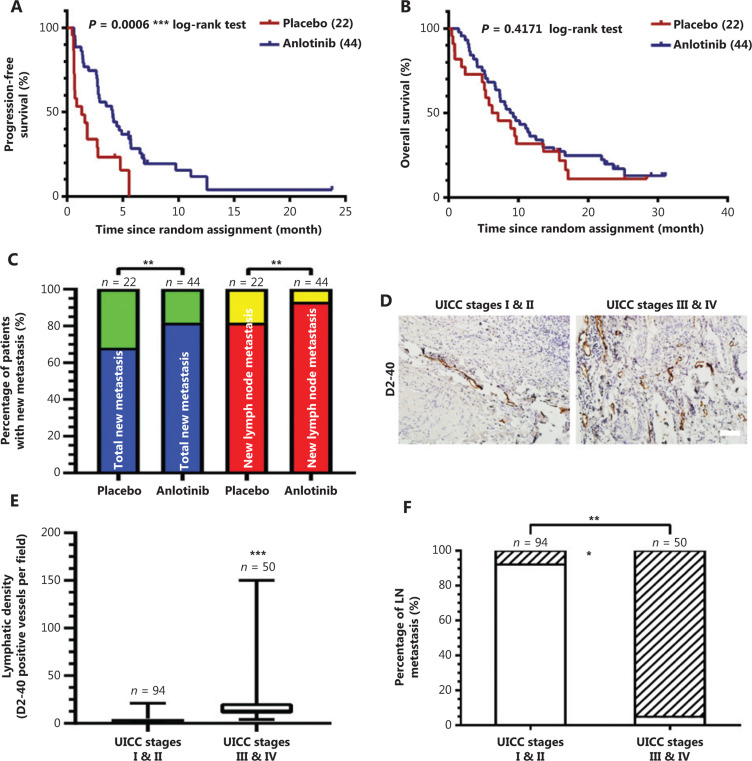
Anlotinib inhibits new metastatic lesions in 44 patients with advanced lung adenocarcinoma. (A) Kaplan-Meier plots of the progression-free survival of patients with advanced lung adenocarcinoma with anlotinib (blue) or placebo (red) treatment (*P* = 0.0006, log-rank test). (B) Kaplan-Meier plots of overall survival in patients with advanced lung adenocarcinoma with anlotinib (blue) or placebo (red) treatment (*P* = 0.4171, log-rank test). (C) Percentages of total new metastatic lesions and new lymph node metastasis of patients with advanced lung adenocarcinoma in the placebo group or anlotinib group. Marks on bars indicate the number of patients in each group (*n* = 22 in the placebo group, *n* = 44 in the anlotinib group). Blue: patients without total new metastatic lesions, green: patients with total new metastatic lesions, red: patients without new lymph node metastasis, yellow: patients with new lymph node metastasis. Spearman rank correlation test, **P < 0.01. (D) Typical images of D2-40 (brown) immunostaining of lymphatic vessels on lung cancer specimens, on the basis of UICC staging. Magnification, 200×, scale bar, 100 μm. (E) Box plot analysis of the correlation between baseline lymphatic density (D2-40 positive vessels per 200× field) and the UICC stages of lung adenocarcinoma patients (*n* = 94 in UICC stages I and II group, *n* = 50 in the UICC stage III and IV group). Data are mean values ± SD. ***P < 0.001; Student’s *t*-test. (F) Percentages of lymph node (LN) metastasis in UICC stages I and II or UICC stages III and IV. Marks on bars indicate the number of patients in each group (*n* = 94 in the UICC stage I and II group, *n* = 50 in the UICC stage III and IV group). White: patients with cancer cell-negative lymph nodes, stripes: patients with cancer-cell positive lymph nodes. Spearman rank correlation test, ***P < 0.001.

We analyzed a subset of patients with advanced lung adenocarcinoma who had been enrolled in the ALTER-0303 study and found a significant difference in progression-free survival between the placebo and anlotinib groups (**Figure 1A**), whereas there was no statistical difference in the overall survival rate (**Figure 1B**). In addition, the rate of new metastatic lesions in the anlotinib group was 18.18%, a proportion almost 2 times lower than that in the placebo group (31.82%) (**Figure 1C**). Moreover, the proportion of patients with new lymph node metastasis was 6.8% in the anlotinib group but 18.2% in the placebo group (**Figure 1C**). The clinical characteristics of 66 patients with lung adenocarcinoma are shown in **Supplementary Table S1**. Because lymphatic vessels in tumors play an essential role in lung cancer metastasis^9^, we measured lymphatic vessel density in 144 clinical specimens of lung adenocarcinoma at various stages by immunohistochemistry, using the lymphatic endothelial cell marker D2-40 (**Figure 1D**). D2-40 expression was positive in most lung cancer specimens. As expected, the lymphatic vessel density increased significantly as the disease progressed, on the basis of Union for International Cancer Control (UICC) stage, and the median baseline lymphatic vessel density values for stages I and II, and stages III and IV were 4.2 and 20.2, respectively (**Figure 1E**). We found that the lymphatic vessel density in lung cancers lesions strongly correlated with lymph node metastasis (**Figure 1F**). Moreover, we examined the correlation between lymphatic vessel density and a series of clinicopathologic parameters in these cases. There were no statistically significant differences in lymphatic vessel density with respect to age, sex and smoking status, but a strong correlation with distant and lymph node metastasis was observed (**Supplementary Table S2**). Given that the lymphatic vessel density is much higher in advanced lung adenocarcinoma than in early disease, and the lymphatic vessel density in lung adenocarcinoma patients is strongly correlated with disease progression, lymph node metastasis, poor prognosis, and surgery not being possible, the analysis of our clinical data showed that anlotinib can inhibit new metastatic lesions of advanced lung adenocarcinoma.

These findings indicate that anlotinib may inhibit new metastasis to some extent *via* suppressing lymphangiogenesis in lung tumors.

### Anlotinib inhibits primary tumor growth and tumor-associated lymphangiogenesis in A549^*EGFP*^ xenograft tumors

**Figure 2:**
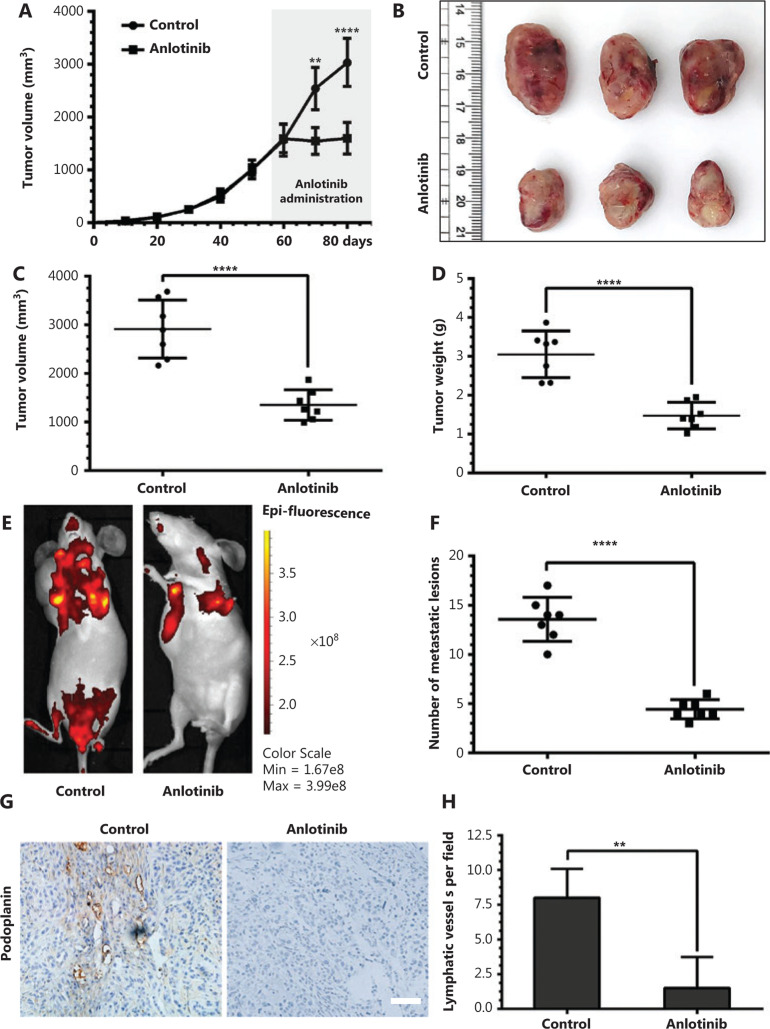
Anlotinib inhibits primary tumor growth and tumor-associated lymphangiogenesis in A549^EGFP^ xenograft tumors. (A) Human lung adenocarcinoma A549^EGFP^ cells were inoculated in the subcutaneous space near the axillary lymph nodes in nude mice, and tumor volume was continuously monitored. (B) Typical images of the primary tumors that formed in mice grafted with human A549^EGFP^ tumor cells on the right shoulder, which were treated with anlotinib or saline. Ruler unit, cm. (C) Scatter plot analysis of volumes of primary tumors treated with anlotinib or saline (*n* = 7 per group). (D) Scatter plot analysis of weights of primary tumors treated with anlotinib or saline (*n* = 7 per group). (E) Representative images of metastatic lesions in different groups of tumor-bearing mice, which were monitored with the IVIS spectrum imaging system. Color bar represents radiant efficiency [(p/s/cm^2^/sr)/(μW/cm^2^)]. (F) A scatter plot analysis showing nearly 3-fold fewer metastatic lesions in the anlotinib group than the control group. (G) Representative images (brown) of podoplanin immunostaining of lymphatic vessels in primary tumors from mice receiving anlotinib or saline treatment. Magnification, 200×, scale bar, 50 μm. (H) Average lymphatic vessel density, as quantified by assessment of the numbers of podoplanin-positively stained vessels in primary tumors of mice (*n* = 7 per group). Data are mean values ± SD. **P < 0.01, ****P < 0.0001; Student’s* t*-test. These experiments were performed 2 times.

To evaluate the effect of anlotinib on human lung adenocarcinoma, we injected A549^EGFP^ cells in the subcutaneous space near the axillary lymph nodes in nude mice for 60 days, then treated the mice with anlotinib or saline for 21 days and continuously monitored tumor volume (**Figure 2A**) (*n* = 7). A substantial inhibition of primary tumor growth was observed in the anlotinib-treated group (**Figure 2B**) (*n* = 7). The median tumor volume in the control group was more than 2 times that in the anlotinib group (**Figure 2C**). In addition, the median tumor weight was approximately 2.1-fold lower in the anlotinib group than the control group (**Figure 2D**). Metastatic lesions were detected by stereomicroscopic imaging, which exhibited a decrease from approximately 15 on average per mouse in the control group to 5 in the anlotinib group (**Figure 2E and 2F**). To determine the effect of anlotinib on lymphatic vessel densities, we examined podoplanin^+^-lymphatic vessels. We performed immunohistochemical staining of podoplanin protein in the primary tumors in these 2 groups and found that the amount of podoplanin^+^ lymphatic vessels was markedly lower in the anlotinib group than the control group (**Figure 2G**). Lymphatic vessel densities (podoplanin^+^) were significantly lower in the anlotinib group than the control group, and the median values were 2 and 7 per field, respectively (**Figure 2H**).

These findings indicate that anlotinib inhibits tumor neolymphangiogenesis *in vivo*.

### Anlotinib decreases tumor colonization in draining lymph nodes

**Figure 3:**
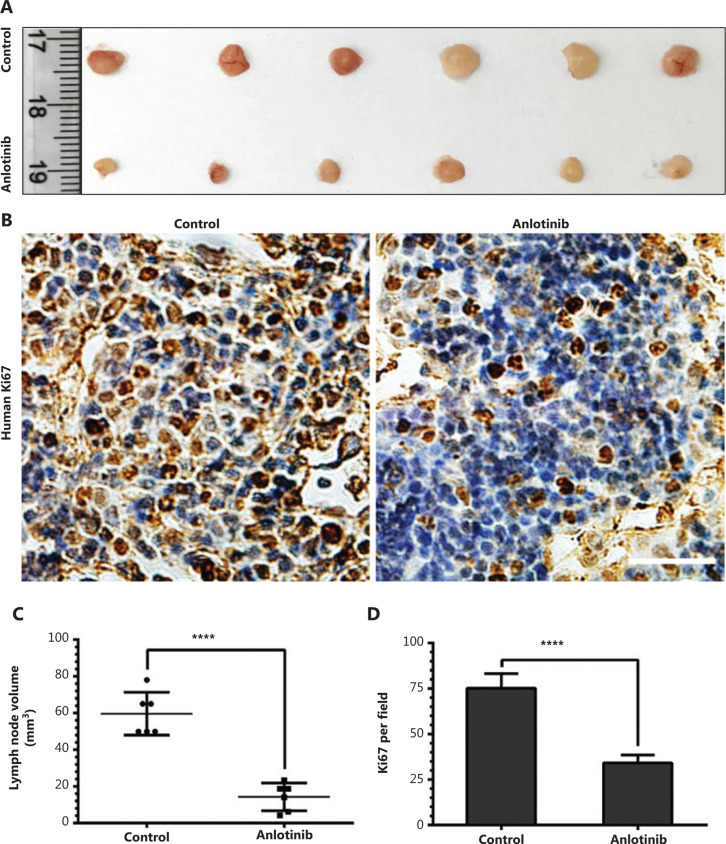
Anlotinib decreases tumor colonization in draining lymph nodes. (A) Typical images of inguinal lymph nodes from different groups, which were treated with anlotinib or saline. Ruler unit, cm. (B) Scatter plot analysis of volumes of lymph nodes treated with anlotinib or saline (*n* = 7 per group). (C) Images of A549^EGFP^ cells positively stained (brown) for human Ki67. Magnification, 400×, scale bar, 50 μm. (D) Number of Ki67-positive A549^EGFP^ cells (*n* = 7 per group) in different groups. Data are mean values ± SD. ****P < 0.0001; Student’s *t*-test. These experiments were performed twice.

Because tumor lymphangiogenesis provides an important route for lymphatic metastasis, we next determined whether anlotinib might prohibit tumor lymph node metastasis. Inguinal lymph nodes, which were tumor draining lymph nodes, were resected after the mice were sacrificed. Autopsy analysis showed that most of the tumor-bearing mice in the control group developed metastatic lesions in inguinal lymph nodes (*n* = 7), which also were much larger in volume than those in the anlotinib group (**Figure 3A and 3B**). Ki67, a proliferation marker, was used to evaluate tumor metastasis. Ki67 staining of lymph nodes confirmed the metastatic lesions (**Figure 3C**), and the analysis of primary tumor metastasis to inguinal lymph nodes (as quantified by the area of human Ki67-positive signal, determined by using anti-human Ki67 antibody, which does not cross react with mouse Ki67) showed an approximately two-fold lower rate of metastasis in the anlotinib group than the control group (**Figure 3D**).

Collectively, these results provide compelling evidence that anlotinib blocks tumor cell colonization in draining lymph nodes.

### Anlotinib inhibits tumor growth and lymphangiogenesis in syngeneic LLC tumors

**Figure 4:**
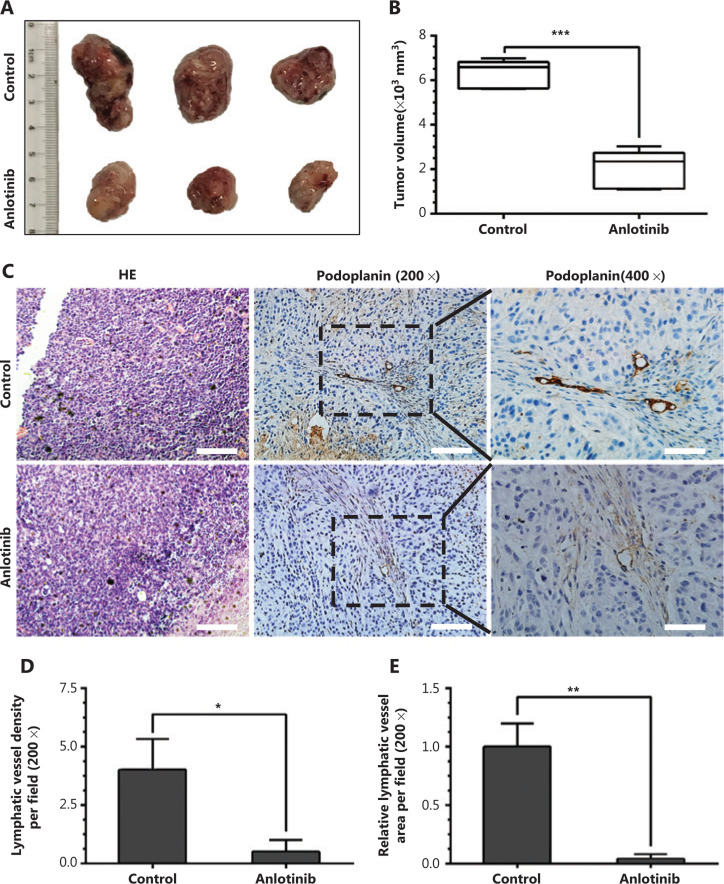
Anlotinib inhibits tumor growth and lymphangiogenesis in syngeneic LLC tumors. (A) Typical images of the primary tumors that formed in mice grafted with LLC tumor cells at the right shoulder, which were treated with anlotinib or saline. Ruler unit, cm. (B) Box plots of volumes of primary tumors (*n* = 6 per group). (C) Representative images (brown) of podoplanin immunostaining of lymphatic vessels in primary tumors in mice treated with anlotinib or saline. Line one: HE-stained cross-sections of the primary tumors from the control or anlotinib group. Magnification, 200×; line two: magnification, 200×, scale bar, 100 μm; line three: magnification, 400×, scale bar: 50 μm. (D) Average lymphatic vessel density, as quantified by assessment of the numbers of podoplanin-positively stained vessels in primary tumors of mice (*n* = 6 per group). (E) Computerized measurements of relative lymphatic vessel area in primary tumors in mice (*n* = 6 per group). Data are mean values ± SD. *P < 0.05, **P < 0.01, ***P < 0.001; Student’s *t*-test. These experiments were performed twice.

To evaluate the influence of anlotinib on lymphangiogenesis in lung cancer, we implanted mouse LLCs in the flanks of C57BL/6J mice to establish a mouse subcutaneous model of lung cancer. A substantial inhibition of primary tumor growth was observed in the anlotinib-treated group (**Figure 4A**). The median tumor volume in the anlotinib group was less than 2 times that of the control group (**Figure 4B**). To determine the effect of anlotinib on lymphatic vessel density, we examined podoplainin expression. We performed immunohistochemical staining of the podoplanin protein in primary tumors in the 2 groups and found that the expression of podoplanin, a marker associated with lymphangiogenesis, was markedly lower in the anlotinib group than the control group (**Figure 4C**). The lymphatic vessel density (podoplanin^+^) was significantly lower in the anlotinib group than the control group, and the median values were 1 and 4 per field, respectively (**Figure 4D**). Furthermore, the lymphatic area decreased approximately a 5-fold in primary tumors in mice administered anlotinib (**Figure 4E**).

These findings indicate that anlotinib inhibits neolymphangiogenesis *in vivo*.

### Anlotinib impairs lymphangioma development via inhibiting neolymphangiogenesis

**Figure 5:**
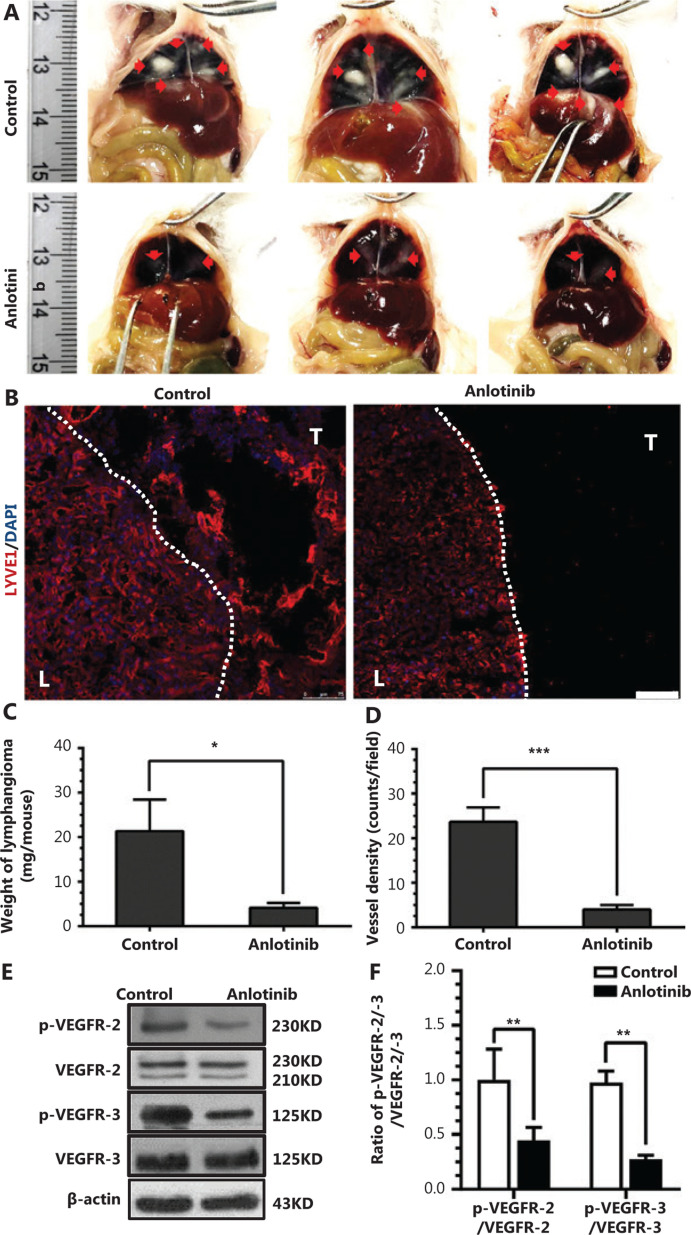
Anlotinib impairs lymphangioma development *via* inhibiting neolymphangiogenesis. (A) Typical images of the benign lymphangiomas (red arrows) that formed in the control and anlotinib groups. Ruler unit, cm. (B) Fluorescence confocal microscopy images showing lymphatic vessels (LYVE1^+^ in tumor area) in frozen sections of lymphangioma tissues from the control and the anlotinib groups, as assessed by immunostaining. L = liver, T = tumor (benign lymphangioma). Red (liver sinusoids were also positive), LYVE1. Blue, DAPI. The dotted line indicates the border between liver and tumor tissues; scale bar, 75 μm. (C) Weights of lymphangioma tissues in the control and anlotinib groups (*n* = 6 per group). (D) Average lymphatic vessel densities, determined by computer-assisted image analysis, of lymphangioma tissues in the livers of anlotinib treated and control mice (*n* = 6 per group). (E) Western blot analysis showing the effects of anlotinib on VEGFR-2 and VEGFR-3 phosphorylation in lymphangioma. (F) Densitometry analysis of VEGFR-2 phosphorylation, VEGFR-2, VEGFR-3 phosphorylation and VEGFR-3 bands in E. Data are mean values ± SD (*n* = 6). *P < 0.05; ***P < 0.001; Student’s *t*-test. These experiments were performed 3 times.

The effect of anlotinib on lymphatic vessel growth was examined *in vivo* in a neolymphangiogenesis model. Intraperitoneal injection of IFA resulted in the development of benign lymphangiomas in the form of opaque plaques (red arrows) with clear boundaries on the liver and diaphragm surfaces of mice (**Figure 5A**). Under experimental conditions, anlotinib treatment, compared with the control, resulted in considerably fewer and smaller plaques; the median weight of lymphangioma plaques was 77.5% lower in anlotinib-treated than control mice (**Figure 5C**). Lymphatic vessel density in cross-sections of lymphangiomas immunostained for LYVE-1 decreased from approximately 24 to 4 counts/mm^2^ (**Figure 5B and 5D**). Western blot analysis of these lymphangiomas indicated that phosphorylation of VEGFR-2 and VEGFR-3 decreased by 2.5-fold and 2.8-fold as a result of anlotinib treatment (**Figure 5E and 5F**).

These findings indicate that anlotinib impairs neolymphangiogenesis *in vivo* by inducing VEGFR-2 and VEGFR-3 dephosphorylation.

### Anlotinib blocks lymphatic tube formation and migration of hLECs *in vitro*

**Figure 6:**
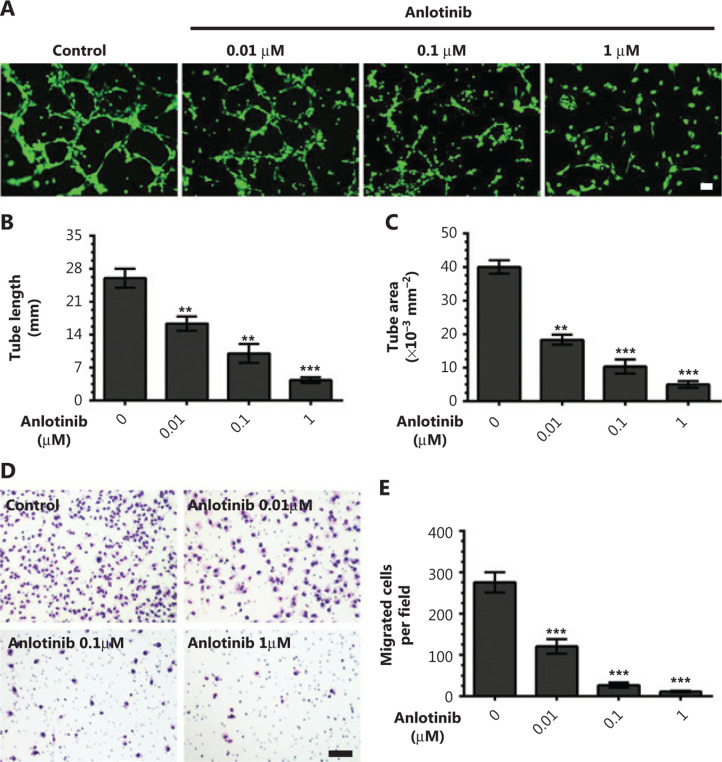
Anlotinib blocks tube formation and migration of hLECs *in vitro*. (A) Typical images of capillary-like tubules formed by hLECs on Matrigel in the absence (control group) or presence (anlotinib group) of different anlotinib concentrations. Magnification, 100×, scale bar, 100 μm. (B) Tube length was significantly shorter in the presence of different anlotinib doses than in the control. (C) Tube area significantly decreased in the presence of different anlotinib doses, as compared with the control. (D) Representative images of the invaded hLECs across a Transwell chamber after 24 h under various experimental conditions. Magnification, 50×, scale bar, 100 μm. (E) Changes in the number of migrated hLECs. Data are mean values ± SD. **P < 0.01, ***P < 0.001; one-way ANOVA. These experiments were performed twice.

To investigate how anlotinib affects the lymphangiogenic process, we performed a series of *in vitro* assays. First, we subjected hLECs to different anlotinib concentrations for IC_50_ value calculations. We found that hLECs were more susceptible to anlotinib (IC_50_ = 2.093 μM, **Supplementary Figure S1**) than either A549^EGFP^ cells (IC_50_ = 14.72 μM, **Supplementary Figure S2**) or LLC cells (IC_50_ = 22.92 μM, **Supplementary Figure S3**). These data prompted us to test anlotinib in different lymphangiogenesis models. We examined capillary tube formation by hLECs on a Matrigel coating and found that anlotinib blocked lymphatic vessel formation in a dose-dependent manner (**Figure 6A**). The tube length and tube area of the lymphatic vessels were lower in the anlotinib group than the control group, and marked inhibition of tube formation was observed when the drug was used at 1 μM (**Figure 6B and 6C**). Finally, by using a Transwell assay to measure the number of hLECs passing across a filter under experimental conditions (**Figure 6D**), we found that anlotinib resulted in lower hLEC migration capacity than the control treatment (**Figure 6E**).

These findings indicated that anlotinib blocked hLEC tube formation and migration.

### Anlotinib interferes with VEGF-C-mediated activation of VEGFR-3 and downstream mediators in hLECs

**Figure 7:**
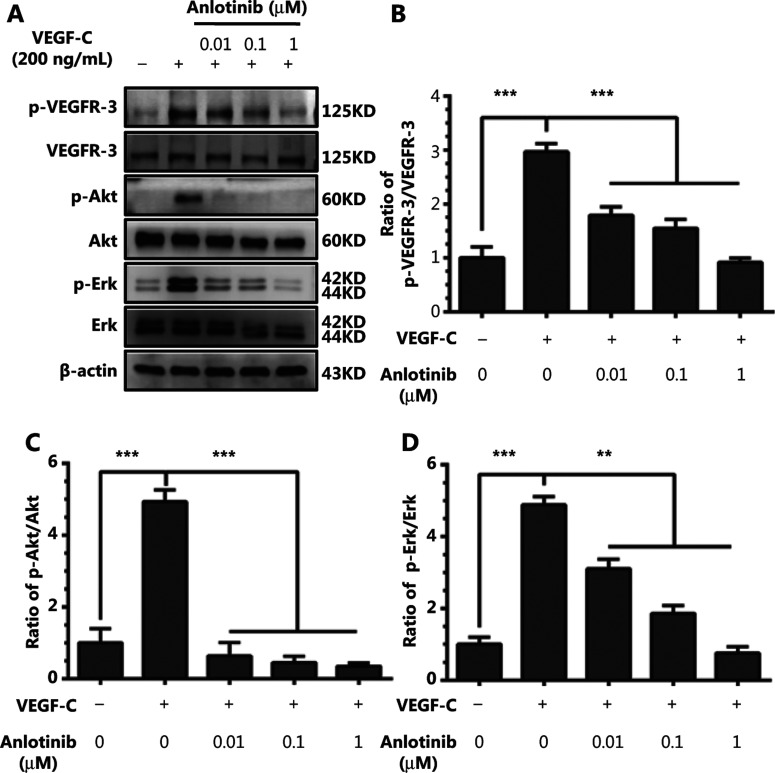
Anlotinib interferes with VEGF-C-mediated activation of VEGFR-3 and downstream mediators in hLECs. (A) Western blot analysis showing the effect of anlotinib on VEGFR-3, Akt and Erk phosphorylation after stimulation with VEGF-C (200 ng/mL). (B) Densitometry analysis of VEGFR-3 phosphorylation and VEGFR-3 bands in A. (C) Densitometry analysis of Akt phosphorylation and Akt bands in A. (D) Densitometry analysis of Erk phosphorylation and Erk bands in a. Data are mean values ± SD. **P < 0.01, ***P < 0.001; one-way ANOVA. These experiments were performed 3 times.

To determine the effect of anlotinib on VEGFR-3 phosphorylation levels, we pretreated hLECs with anlotinib at different concentrations (0, 0.01, 0.1, and 1 μM) and then stimulated these hLECs with VEGF-C (200 ng/mL). VEGF-C treatment resulted in a considerable increase in VEGFR-3 phosphorylation levels; Erk and Akt, downstream factors of VEGFR-3, were also activated. In this experiment, even low doses of anlotinib (0.01 μM) inhibited phosphorylation of VEGFR-3 and downstream mediators (**Figure 7A**). Clear changes in the levels of VEGFR-3 phosphorylation under VEGF-C and anlotinib treatment were observed (**Figure 7B**). Changes in the levels of Akt phosphorylation under VEGF-C and anlotinib treatment were also notable (**Figure 7C**). However, changes in the levels of Erk phosphorylation under VEGF-C and anlotinib treatment were similar (**Figure 7D**).

These data indicate that anlotinib exerts its inhibitory function through interfering with VEGFR-3 signaling pathway activation.

### Schematic presentation of the process involved in lymphatic metastasis suppressed by anlotinib

**Figure 8:**
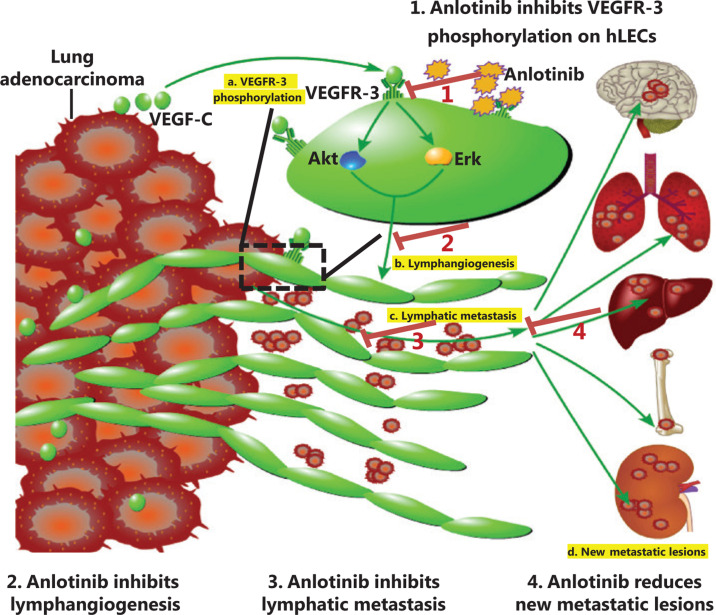
Schematic presentation of suppression of lymphatic metastasis by anlotinib. VEGF-C binds its receptor VEGFR-3 on hLECs (process a), thus causing the phosphorylation of Akt and Erk, then promoting lymphangiogenesis (process b), lymphatic metastasis (process c), and formation of new distant metastatic lesions (process d). These 4 processes are suppressed by anlotinib (steps 1, 2, 3 and 4, respectively).

VEGF-C binds its receptor VEGFR-3 on hLECs (**Figure 8**, process a), thus causing the phosphorylation of Akt and Erk, and subsequently promoting lymphangiogenesis (**Figure 8**, process b), lymphatic metastasis (**Figure 8**, process c), and the formation of new distant metastatic lesions (**Figure 8**, process d). These 4 processes are suppressed by anlotinib (**Figure 8**, steps 1, 2, 3 and 4, respectively).

## Discussion

Anlotinib, a novel oral multi-target receptor tyrosine kinase inhibitor, has become a new third-line treatment option for refractory advanced NSCLC^18,19^. As we have shown, anlotinib decreased the ratio of new metastatic lesions in patients with advanced lung adenocarcinoma and prolonged their progression-free survival. In addition, D2-40 positive lymphatic vessels in 144 Chinese patients with lung adenocarcinoma had low-level density at UICC stages I and II and high level density at UICC stages III and IV. On the basis of these clinical events, we propose the following hypothesis: lymphangiogenesis might play a role in new metastases, and anlotinib may decrease metastases partly *via* inhibition of lymphangiogenesis in advanced lung adenocarcinoma.

Previous studies have indicated that anlotinib inhibits tumor growth *via* anti-angiogenic effects^20,22,23^. However, the effect of anlotinib on tumor lymphangiogenesis and the underlying mechanism were unknown. Here, we demonstrate that anlotinib inhibits hLEC migration and tube formation *in vitro* and blocks lymphangiogenesis in benign lymphangioma. Furthermore, anlotinib inhibits tumor lymphangiogenesis and lymphatic metastasis in lung cancer. Mechanistically, anlotinib abrogates the activation of the VEGFR-3 signal, which is induced by VEGF-C. Subsequently, phosphorylation of molecules downstream of VEGFR-3, such as Erk and Akt, is also suppressed after anlotinib treatment.

VEGFR-3 is expressed in lymphatic endothelial cells^26^, tumor-associated macrophages^27^, and monocytes^28^. VEGFR-3 plays a crucial role in lymphangiogenesis, and inhibition of VEGFR-3 signaling suppresses tumor lymphangiogenesis and lymph node metastasis in a variety of tumor models^10,29^. The experimental data from the present study indicate that anlotinib-mediated abrogation of VEGFR-3 and subsequent activation of its downstream effectors (i.e., Erk and Akt^30^) in LECs blocks lymphangiogenesis during benign lymphangioma development and tumor progression through lymphatic vessels, because the incidence of lymph node metastasis was decreased by anlotinib treatment in a mouse model of lung cancer. Our findings suggest that anlotinib may play an important role in the regulation of lymphangiogenesis and may be useful in suppressing systemic dissemination of cancer.

The concentration of anlotinib used in the present study was determined mainly through pre-tests in different cell lines, wherein the lowest inhibitory IC_50_ (IC_50_ = 2.093 μM) was found in hLECs among the 3 cell types, thus indicating that hLECs may be more sensitive to anlotinib. These results suggest the promise of anlotinib treatment in abrogating tumor lymphangiogenesis *in vivo*. In a clinical pharmacokinetic study, the plasma concentration of anlotinib reached 100 ng/mL (0.21 μM) after patients were treated with 12 mg/kg of oral anlotinib daily for 14 days, which is the typical therapeutic dosage. According to a rough conversion, the minimum effective concentrations in our cell experiments (0.01 μM for hLECs and 0.1 μM for A549^EGFP^ cells) and in animal experiments (3 mg/kg) are even lower than the standard clinical dose.

Additionally, Erk- and Akt-associated drug resistance can develop even when patients are treated with the standard dose of chemotherapy in a clinical setting^31^. Our study indicated that phosphorylation of Erk and Akt was suppressed by anlotinib treatment in A549^EGFP^ cells (**Supplementary Figure S4**). This finding suggests that anlotinib may be able to overcome Erk- and Akt-dependent resistance.

## Conclusions

In summary, this study reveals that anlotinib suppresses tumor-derived lymphangiogenesis and lymphatic metastasis *via* targeting the VEGFR-3 signaling pathway in lung adenocarcinoma (**Figure 8**). First, our study confirmed that the new oral drug anlotinib can decrease new metastatic lesions. Second, in 44 patients with advanced lung adenocarcinoma, we found that the D2-40^+^-lymphatic vessel density was much higher in advanced than early stage lung adenocarcinoma in 144 patients with lung adenocarcinoma. On the basis of these findings, we propose that anlotinib partly inhibits new metastatic lesions through inhibiting lymphangiogenesis in patients with advanced lung adenocarcinoma. Second, our findings were consistent with our hypothesis that anlotinib would inhibit tumor lymphangiogenesis and lymphatic metastasis in various mouse models. Finally, mechanistically, we determined that this effect is mediated by VEGFR-3 both in benign lymphangioma tissues and in lymphatic endothelial cells. Our findings suggest that anlotinib may be beneficial in the treatment of lung cancer by suppressing lymphangiogenesis and lymphatic metastasis.

This study has several limitations. Anlotinib is a multi-target drug that can affect a variety of tyrosine kinases. It inhibits tumor metastasis not only through lymphatic metastasis but also through blood metastasis. Which form of tumor metastasis is more strongly inhibited by anlotinib and which molecule (VEGFR-2 or VEGFR-3) plays a more important role in tumor metastasis remain to be determined.
