# Supplementary material for: Anlotinib suppresses lymphangiogenesis and lymphatic metastasis in lung adenocarcinoma through a process potentially involving VEGFR-3 signaling
Source: Cancer Biol Med. 2020 Aug 15;17(3):753–67. doi: 10.20892/j.issn.2095-3941.2020.0024 (PMC7476093; doi:10.20892/j.issn.2095-3941.2020.0024)
Supplement: Supplementary file 1 [file cbm-17-753-s001.pdf]

Supplementary materials

Table S1 Clinical characteristics of 66 patients with lung adenocarcinoma

Characteristics	Cases	Group		*P*
		Placebo, *n*	Anlotinib, *n*	
Sex				0.481
Male	38	14	24	
Female	28	8	20	
Age, years				0.116
≤ 60	36	9	27	
> 60	30	13	17	
Smoking				0.444
Yes	28	7	21	
No	38	15	23	
TNM stage				0.466
IIIB	4	2	2	
IV	62	20	42	
No. of baseline metastatic lesions				0.862
≤ 3	35	12	23	
> 3	31	10	21	
No. of patients with new metastatic lesions				< 0.01**
0	51	15	36	
≥ 1	15	7	8	
No. of patients with new lymph node metastatic lesions				< 0.01**
0	59	18	41	
≥ 1	7	4	3	
ECGO performance				0.29
0	9	1	8	
1	55	20	35	
2	2	1	1	

EGFR, epidermal growth factor receptor; ECGO, Eastern Collaborative Oncology Group; *P*-value were calculated with Spearman rank correlation test. ***P* < 0.05.

Table S2 Correlation between lymphatic vessel density (lymphangiogenesis) and clinicopathologic characteristics in 144 patients with lung adenocarcinoma

Characteristics	Cases	Lymphatic vessel density		*P*
		Mean	SEM	
Sex				0.057
Male	69	11.75	18.896	
Female	75	8.80	13.270	
Age, years				0.514
≤ 60	87	9.20	12.599	
> 60	57	11.77	20.592	
Smoking				0.855
Yes	78	10.49	19.164	
No	66	9.99	13.359	
M stage				< 0.001***
M0	113	9.51	16.362	
M1	11	18.73	11.917	
N stage				< 0.0001****
N0	90	5.06	5.481	
N1	12	9.50	4.622	
N2	41	21.89	25.830	
N3	1	4.00	/	
T stage				< 0.0001****
T1	80	6.84	11.611	
T2	42	15.46	24.067	
T3	15	12.63	7.640	
T4	7	12.14	6.122	
TNM stage				< 0.0001****
I	76	3.79	3.914	
II	18	8.08	4.784	
III	37	21.66	26.720	
IV	13	18.15	11.157	

P value were calculated with Spearman rank correlation test; ****P* < 0.001; *****P* < 0.0001.

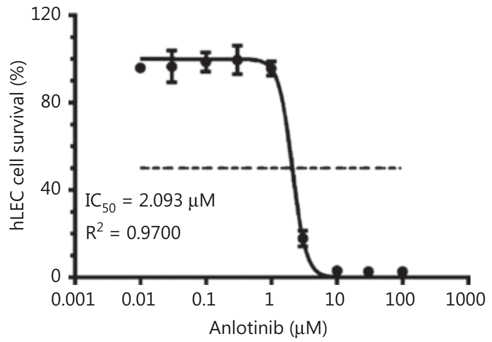

Figure S1 Anlotinib inhibits hLEC cell proliferation.

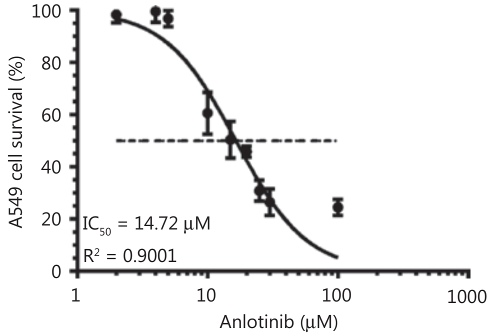

Figure S2 Anlotinib inhibits $A549^{EGFP}$ proliferation.

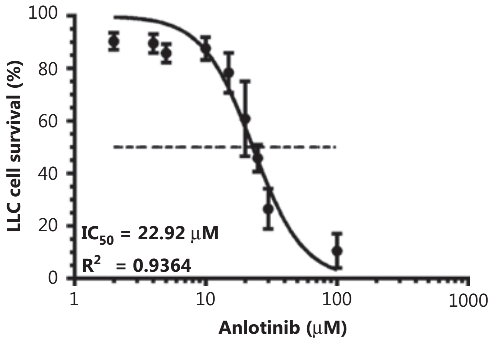

Figure S3 Anlotinib inhibits LLC proliferation.

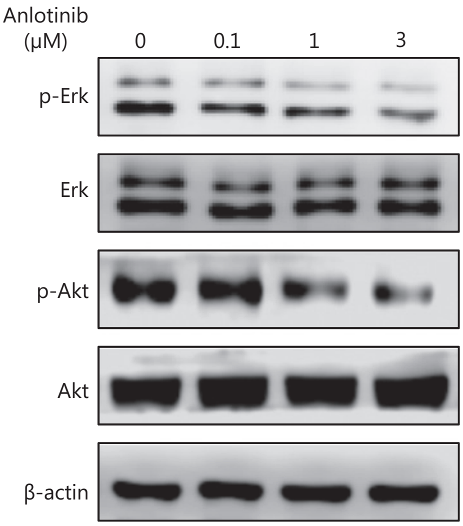

Figure S4 Anlotinib inhibits phosphorylation of Akt and Erk in A549^{EGFP} cells.
